# From chemical composition to clinical application: the value of bile-based traditional Chinese medicines in the treatment of liver diseases

**DOI:** 10.3389/fphar.2026.1789551

**Published:** 2026-04-24

**Authors:** Xue Liang, Yuehui Liang, Siqi Liu, Lin Yu, Yunxiao Liang, Lei Zhou

**Affiliations:** Department of Gastroenterology, Institute of Digestive Disease, Guangxi Academy of Medical Sciences, The People’s Hospital of Guangxi Zhuang Autonomous Region, Nanning, China

**Keywords:** bile acid receptor, bile acids, bile-based traditional Chinese medicine, clinical applications, liver diseases

## Abstract

Bile-based traditional Chinese medicines comprise a special category of medicinal materials that have distinct value in the treatment of liver diseases. Bile acids are the primary active components of bile-based TCMs. These components exert significant therapeutic effects by targeting key signaling pathways related to disease progression through bile acid receptors such as FXR and TGR5. Herein, we comprehensively review the chemical composition, pharmacological effects, and clinical applications of bile-based TCMs with a particular focus on their potential and recent research progress in the treatment of liver diseases. The bile acid components of bile-based TCMs have dual “Yin and Yang” effects in the treatment of liver diseases. They show therapeutic actions such as anti-inflammation, hepatoprotection and anti-fibrosis, and they may also promote disease through certain constituents like TCA and GCA. This dual nature creates a complex balance between benefit and risk. It highlights the mechanistic complexity of bile-based TCMs in treating liver diseases and presents new challenges for their precise clinical application. Future research needs to thoroughly analyze the composition and efficacy of bile-based TCMs, clarify their mechanisms of action and pharmacological effects, and explore component modification and combination therapies. This will provide a scientific basis and innovative ideas for precise application of bile-based TCMs and development of new, more effective and safer treatment options for patients with liver diseases.

## 1 Introduction

Bile-based traditional Chinese medicines (TCMs) trace back over a millennium. The *Compendium of Materia Medica* describes bear bile as an agent to clear liver fire and resolve heat toxin, and it is found in hundreds of classical formulations. Traditional experience now faces an unprecedented public health challenge. Chronic liver disease causes about two million deaths each year, accounting for four percent of global mortality. The prevalence of metabolic dysfunction-associated steatotic liver disease (MASLD, formerly NAFLD) exceeds 30%, and 30% of related hepatocellular carcinoma can appear without cirrhosis, indicating a large gap for early intervention (Gan et al., 2025). Available therapies remain constrained. Nucleoside analogues can suppress hepatitis B virus replication for years, but cccDNA persists, with a functional cure rates of less than 10%, and resistance and drug resistance and nephrotoxicity will occur over time (Perrillo, 2005). In Metabolic dysfunction-associated steatohepatitis (MASH), only resmetirom has been approved by the U.S. Food and Drug Administration, and its indication is narrow. Obeticholic acid (OCA) and other phase three candidates have terminated development due to hepatotoxicity or insufficient efficacy (Unagolla et al., 2024). Most available drugs target a single molecule and fail to regulate the gut-liver axis comprehensively, preventing simultaneous correction of metabolic disorder, inflammation and immune imbalance. An urgent demand exists for safe, broadly available therapies with multi-target capabilities. Bile-based TCMs contain abundant bile acids that regulate Farnesoid X receptor (FXR), Takeda G protein-coupled receptor 5 (TGR5) and other pathways, and their “Yin-Yang” actions offer a new direction for solving these problems.

Beyond their pharmacological roles, bile acids are integral to systemic nutritional homeostasis (Boyer, 2013). As primary cholesterol metabolites, they act as digestive surfactants that facilitate the emulsification and absorption of dietary lipids and fat-soluble vitamins (A, D, E, and K) in the small intestine (Staels et al., 2007). Bile-based TCMs, which are often obtained as bioactive by-products of the food and livestock industry, represent a unique bridge between dietary metabolism and clinical therapy (Li et al., 2016). By modulating the gut-liver axis and regulating glucose and lipid sensors, these substances offer a potential nutritional intervention for diet-induced metabolic disorders like MASLD and obesity (Gan et al., 2025; Kuang et al., 2023).

**FIGURE 1:**
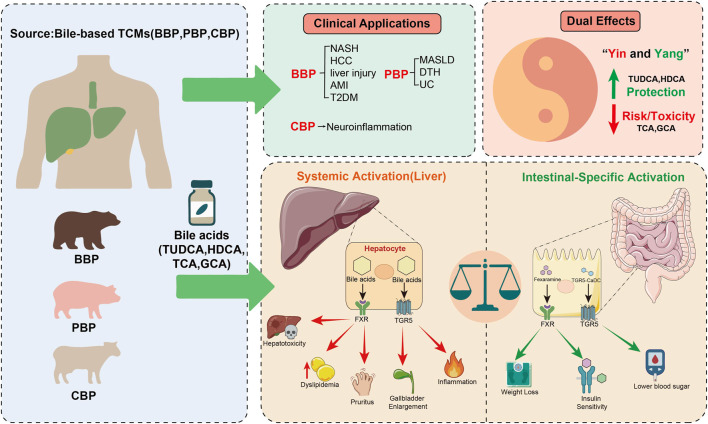
Roadmap of bile-based TCMs: from natural sources to precision receptor modulation. This model integrates the core narrative of the review. Sources and Applications: Bile powders (BBP, PBP, CBP) provide bioactive bile acids for treating various metabolic and inflammatory liver conditions. The “Yin-Yang” Dualism: This framework balances therapeutic protection (TUDCA, HDCA) against potential toxicity risks (TCA, GCA). Targeting Strategy: The model contrasts the side effects of systemic hepatic activation (e.g., pruritus, hepatotoxicity) with the benefits of intestinal-specific activation. Transitioning toward tissue-restricted agonists represents a precision medicine strategy to maximize metabolic efficacy while minimizing systemic risks.

The primary objective of this review is to bridge the gap between traditional TCM theory and modern molecular pharmacology. We focus on bile acids as key pharmacological entities and evaluate how their interactions with receptors, such as FXR and TGR5, explain the dual nature of bile-based TCMs. This review provides a conceptual framework based on the “Yin-Yang” balance, where therapeutic efficacy and potential toxicity are determined by bile acid composition and activation thresholds. By integrating clinical evidence, including comparisons between traditional bile powders and modern standards like TAF, with receptor biology, we aim to propose new strategies for tissue-specific drug delivery. This systematic approach provides a roadmap for the transition from traditional use to the precision application of bile-based therapies in modern hepatology (Figure 1).

## 2 Classification and components of bile-based traditional Chinese medicines

Bile-based traditional Chinese medicines (TCMs) are an important part of traditional Chinese medicine. These medicines come from the bile or dried gallstones of several animals. After careful processing and drying, they are used in the form of bile powder. Examples include bear bile powder (BBP), pig bile powder (PBP), and cow bile powder (CBP). Ancient herbal texts such as *Compendium of Materia Medica*, *Pharmacopoeia of the People’s Republic of China*, *Collected Annotations on the Classic of Materia Medica*, and *Treatise on Cold Damage Disorders* all have detailed records of these bile-based TCMs (Jiansheng et al., 2003). According to TCM theory, animal-derived medicines are considered to be “blood and flesh substances with unique affinity”, due to their special rich smell, and are readily absorbed by the human body. Currently, following more in-depth research, animal-derived drugs are receiving increased attention because of their wide availability, relatively few side effects, and significant therapeutic effects. Bile-based TCMs have broad clinical utility and prospects for treatment of liver diseases.

### 2.1 BBP

BBP is obtained from the dried gallbladder bile of black or brown bears, or other Ursidae species, after a series of processing steps. For thousands of years, it has played an important role in TCM clinical practice (Yingjie et al., 2010; Yanhong et al., 2012). In the classic TCM formula system, more than 396 formulas contain bear bile components as key ingredients (Feng et al., 2009). Modern chemical research has shown that BBP is mainly composed of bile acids, phospholipids, cholesterol, bilirubin, proteins, and inorganic salts (Wang et al., 2011). Among these, bile acids play the main therapeutic role. The bile acids in BBP are all in the form of taurine conjugates, including taurocholic acid (TCA), tauroursodeoxycholic acid (TUDCA), taurodeoxycholic acid (TDCA), and taurochenodeoxycholic acid (TCDCA). These conjugated bile acids are formed by the combination of taurine with each of the corresponding free bile acids. In a survey study, in 8 batches of BBP, the two main components, TUDCA and TCDCA, accounted for 39.04% ± 7.83% and 36.10% ± 9.42%, respectively (Qiao et al., 2014). Ten different samples of BBP purchased from the market were analyzed by ^1^H-nuclear magnetic resonance (NMR) spectroscopy combined with liquid chromatography-mass spectrometry (LC-MS). It was found that the concentration of TUDCA was highest at 568.488 nmol/mg, followed by TCDCA (282.614 nmol/mg), and CDCA (29.074 nmol/mg) (Table 1) (Yan et al., 2022). These findings suggest that the relatively high levels of TUDCA and TCDCA in bear bile distinguish it from other animal-derived bile (Feng et al., 2009; Wang et al., 2011; Wang et al., 2012).

**TABLE 1:** Classification and chemical constituents of bile-based TCMs.

Category of bile-based TCMs	Type of component	Content	Unit	Detection method	Relative content (%)	Detection method	References
BBP	TUDCA	568.488	nmol/mg	^1^H-NMR and LC-MS	39.04 ± 7.83	UHPLC–qTOF-MS	Qiao et al. (2014), Yan et al. (2022)
	TCDCA	282.614	​	​	36.10 ± 9.42	​	​
	CDCA	29.074	​	​	​	​	​
	TCA	24.483	​	​	​	​	​
	UDCA	19.928	​	​	​	​	​
	CA	5.24	​	​	​	​	​
PBP	GHDCA	518.01	nmol/mg	^1^H-NMR and LC-MS	24.6	HPLC-ELSD	Yan et al. (2022), Yan et al. (2017)
	GCDCA	​	​	​	18.4	​	​
	GCA	38.59	​	​	​	​	​
	TCDCA	11.72	​	​	​	​	​
	HDCA	4.53	​	​	​	​	​
	TCA	2.78	​	​	​	​	​
	CA	1.32	​	​	​	​	​
CBP	GCA	510.23	nmol/mg	^1^H-NMR and LC-MS	25.2	HPLC-ELSD	Yan et al. (2022), Yan et al. (2016)
	TCA	413.77	​	​	24.5	​	​
	TCDCA	411.82	​	​	​	​	​
	CA	19.36	​	​	​	​	​
	CDCA	1.79	​	​	​	​	​
	GHDCA	1.38	​	​	​	​	​
	GDCA	​	​	​	4.1	​	​
	TDCA	​	​	​	5.2	​	​
	GUDCA	0.63	​	​	​	​	​

### 2.2 PBP

PBP is obtained from the bile of pigs (family Suidae). It is obtained by collection after slaughter, followed by air drying or pressing and further dehydration processes (Binghong and Jing, 2000). It contains multiple components, including bile acids, bilirubin, proteins, and lipids, with bile acids being the most abundant. The major bile acids found in PBP are hyocholic acid (HCA), hyodeoxycholic acid (HDCA), chenodeoxycholic acid (CDCA), taurohyodeoxycholic acid (THDCA), glycohyodeoxycholic acid (GHDCA), glycochenodeoxycholic acid (GCDCA) and TCDCA (Qihua et al., 2012; Kandrac et al., 2006; He et al., 2012; Zhiwei et al., 2010). Among these, glycine-conjugated bile acids predominate in PBP. Analysis of 20 samples of PBP using high-performance liquid chromatography with evaporative light scattering detection (HPLC-ELSD) found that GHDCA accounted for about 24.6%, GCDCA about 18.4%, with the combined proportion of the two being around 40% (Yan et al., 2017). A study using ^1^H-NMR and LC-MS showed that GHDCA was the most abundant bile acid in PBP at 518.01 nmol/mg, followed by glycocholic acid (GCA, 38.59 nmol/mg) and TCDCA (11.72 nmol/mg) (Table 1) (Yan et al., 2022). These components have important bioactivity and pharmacological effects, informing the clinical use of PBP.

### 2.3 CBP

Bile from bovine animals (family Bovidae) is typically processed by filtration, drying, and pulverization to produce CBP for medicinal use. Bile acids are the most abundant components and include GCA, glycodeoxycholic acid (GDCA), GCDCA, cholic acid (CA), deoxycholic acid (DCA), TCA, TDCA, TCDCA, and CDCA (Qiao et al., 2011). Among these, GCA, TCDCA, and TCA are the main components (Qiming, 1997; Nengrong, 1983), while the concentration of free bile acids is usually very low. Analysis of 13 batches of CBP by HPLC-ELSD showed that GCA and TCA were the most abundant bile acids in cow bile powder, accounting for 25.2% and 24.5%, respectively (Yan et al., 2016). Another study found that GCA was the most abundant component in CBP at 510.23 nmol/mg, followed by TCA (413.77 nmol/mg) and TCDCA (411.82 nmol/mg) (Table 1) (Yan et al., 2022).

## 3 Pharmacological effects and clinical applications of bile-based TCMs

### 3.1 BBP

Bear bile, characterized by its cold and cool properties, has a bitter and cool taste. It can enter the liver, gallbladder, and heart meridians. Its effects include clearing heat and detoxifying, reducing liver fire, and relieving spasms (Chaofan et al., 2015). Modern pharmacological studies in recent decades have confirmed that BBP has pharmacological effects that include hepatoprotection, antibacterial, antiviral, anti-inflammatory, anti-cholelithiasis, and lipid-lowering properties (Jiajing et al., 2016; Daxin, 2018; Liang et al., 2024). In TCM clinical practice, BBP is widely used to treat fever, for detoxification, to reduce inflammation and decrease swelling, and to relieve pain (Feng et al., 2009; Chaofan et al., 2015). It has exhibited particularly strong efficacy in the treatment of liver-related conditions such as hepatic fibrosis, cholestatic cirrhosis, and even hepatocellular carcinoma (HCC), where it reduces liver fire and heat (Zhiru and Jingbo, 2015). In clinical practice, the use of bear bile powder has expanded to other liver conditions such as viral hepatitis, fibrosis, and HCC (Yan et al., 2014; Dutton et al., 2011; Bin et al., 2014; Chunmei et al., 2006; Qingqing et al., 2013; Changsheng et al., 2011; Changsheng et al., 2012; Yan et al., 2013). In traditional medicine, it is also used to treat red and swollen eyes caused by excessive liver fire and heat. Despite its significant therapeutic effects, the specific mechanisms mediating the efficacy of BBP await elucidation. It is currently believed that its hepatoprotective effects are related to its ability to enhance immunity and regulate the microenvironment (Yan et al., 2014; Dutton et al., 2011; Bin et al., 2014; Chunmei et al., 2006; Qingqing et al., 2013; Changsheng et al., 2011; Changsheng et al., 2012; Yan et al., 2013; Guanjun et al., 2014). The specific mechanisms of BBP in regulating the liver microenvironment and immunity are primarily driven by its major bile acid components. For instance, TUDCA has been shown to enhance the phagocytic activity of Kupffer cells (Funaoka et al., 1999), and it suppresses the inflammatory tumor microenvironment by inhibiting NF-κB signaling and downregulating VEGF expression (Kim et al., 2019). TCDCA acts as an anti-inflammatory mediator by binding to the TGR5 receptor on macrophages, which triggers the cAMP-PKA-CREB signaling pathway to reduce the release of pro-inflammatory cytokines like TNF-α, IL-1β, and IL-6 (Qi et al., 2020). Additionally, TCDCA promotes the secretion of IL-10 and inhibits the activation of hepatic stellate cells (HSCs), thereby reducing fibrotic mediator release (Zeng et al., 2025). UDCA contributes to immune regulation by acting on NK and NKT cells via the glucocorticoid receptor (GR) to lower IFN-γ production (Takigawa et al., 2013). It also inhibits ADAM17 activity in biliary epithelial cells, which prevents the excessive release of pro-inflammatory factors (Buryova et al., 2013). However, the microenvironment is also affected by components like TCA, which promotes the activation and proliferation of quiescent HSCs through the S1PR2/p38 MAPK/YAP signaling pathway (Yang et al., 2023). Together, these diverse cellular actions of bile acids explain the systemic effects of BBP on liver immunity and the tissue microenvironment.

Studies have shown that BBP is highly effective for treating liver diseases such as non-alcoholic steatohepatitis (NASH), HCC, and cholestatic liver injury. Studies have shown that Biotransformed bear bile powder (BBBP) ameliorates high-fat-high-sugar-induced NASH in mice by modulating the FXR/PXR-PI3K-AKT-NOS3 axis and reprogramming arginine biosynthesis. Specifically, BBBP alleviates liver steatosis, inflammation and fibrosis, reduces body weight and blood sugar levels, and improves insulin resistance. At the molecular level, the major bile acid components in BBBP (TUDCA, TCA, TCDCA), act as ligands for the nuclear receptors FXR and PXR. These components directly activate the nuclear receptors FXR and PXR, enhancing their interaction with EGFR. This subsequently triggers PI3K-AKT signaling and increases NOS3 transcription and upregulates NOS3. As a key downstream effect molecule of this pathway, NOS3 not only promotes the arginine metabolic cycle, but also improves nitrogen metabolism disorders and alleviates liver lipid deposition and inflammatory responses by regulating the levels of intermediate metabolites in the urea cycle (such as citrulline, ornithine, glutamic acid, etc.). These changes reduce hepatic steatosis, inflammation and fibrosis, lower body weight and fasting glucose, and improve insulin sensitivity. The findings identify an FXR/PXR-driven cross-talk pathway that links lipid and amino-acid metabolism and provide a mechanistic rationale for BBBP in NASH therapy (Jiang et al., 2023). BBP has also shown potential for HCC treatment in recent studies. It significantly reduced tumor volume and weight in HCC mouse models. It promoted cancer cell apoptosis, and inhibited cell proliferation and tumor angiogenesis. Its mechanisms of action were linked to suppression of signal transducer and activator of transcription 3 (STAT3) signaling pathway and regulation of key target gene expression (Chen et al., 2020). Additionally, cultured bear bile powder (CBBP) has been shown to effectively alleviate serum liver injury indicators in mice with cholestasis. It reduced neutrophil infiltration and liver cell necrosis, suppressed the TLR4/MyD88/NF-κB signaling pathway, reducing inflammatory factor expression, and alleviated liver inflammation. It also downregulated apoptosis-related protein expression, reduced liver cell apoptosis, and effectively ameliorated cholestatic liver injury (Cai et al., 2022).

Controlled studies show that bear-bile capsules or injections has a significant therapeutic effect on patients with hepatitis or cirrhosis. Qin et al. enrolled 78 acute or chronic cases infected with hepatitis A, B, E or mixed viruses. After 4 weeks of bear-bile capsule treatment, 87% of subjects reported symptom relief and 81% achieved normal total bilirubin, both higher than the 51% and 40% recorded in the Yigganling control group (*P* < 0.01). The ALT normalisation was 80% in both groups (Shan et al., 2000). Current standard drugs for viral hepatitis include entecavir (ETV) and tenofovir alafenamide fumarate (TAF). ETV treatment for chronic hepatitis B over 48 weeks achieved an ALT normalization rate of 67.8% (Kim et al., 2021). In contrast, TAF treatment maintained an ALT normalisation rate of >93% after 40 months in patients with chronic hepatitis B, with a 100% virological response. Some patients with low HBsAg levels reached functional cure, and TAF showed significantly better bone and renal safety profiles compared to ETV. Consequently, TAF is now recommended as first line therapy (Barr et al., 1985). While indirect data from different studies suggest that bear bile capsules may achieve competitive biochemical response rates compared to early nucleoside analogues, these observations must be interpreted with caution. In the absence of direct head-to-head clinical trials, it is premature to draw definitive conclusions regarding the relative efficacy of bear bile capsules against ETV or TAF. Furthermore, TAF remains the preferred first-line therapy due to its validated long-term virological response and safety. Consequently, the potential role of BBP as an adjuvant therapy requires further validation through rigorous, directly controlled clinical investigations. Additionally, Chen et al. combined bear-bile capsules with Danshen for 86 chronic hepatitis B patients with refractory jaundice (TBil > 50 μmol·L^−1^) and observed significant bilirubin reduction (Ming and Dan, 2010). Shi et al. treated 280 icteric-hepatitis patients with bear-bile injection, with a total effective rate of 96.1%, which was higher than the rates of observed in groups treated with Yinchen decoction (84.3%) and Ganlixin (88.0%) (Lixia et al., 2001). Guo et al. found that serum hyaluronic acid decreased significantly after bear-bile capsule administration in chronic hepatitis B, suggesting anti-fibrotic activity and a possible slowing of cirrhotic progression (Wen and Deyong, 1996). These clinical data corroborate earlier mechanistic findings in animals and strengthen the rationale for translating BBP to clinical use in hepatitis, cholestasis and liver cancer.

**FIGURE 2:**
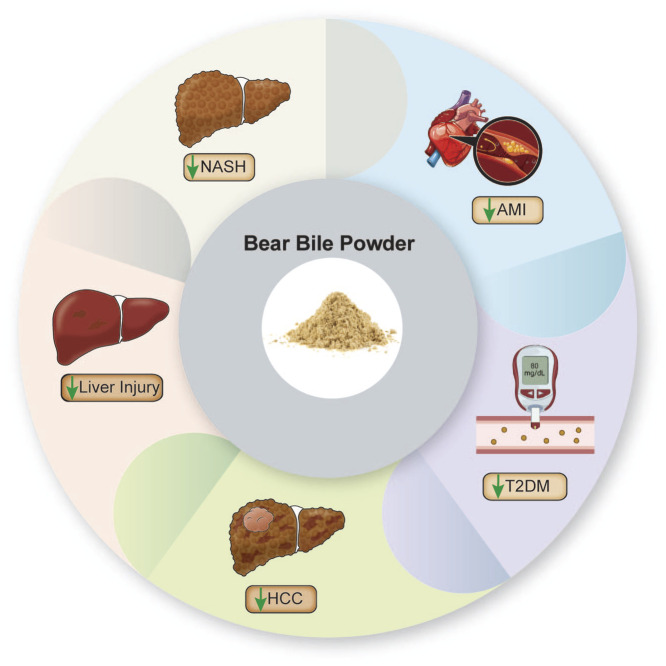
Clinical applications of BBP.

BBP has also shown significant therapeutic effects in the treatment of various non-liver diseases. It is a key component of Shexiang Tongxin Dropping Pills (STDP), which is used clinically for cardiovascular diseases such as heart failure and angina pectoris. In a rat model of acute myocardial infarction (AMI), BBP combined with CF (STDP without BBP) significantly reduced myocardial infarct size and pathological changes. It lowered serum lactate dehydrogenase and creatine kinase levels, and improved heart function. BBP regulated gut microbiota and metabolites, restoring AMI-induced gut microbiota imbalance, thereby alleviating AMI (Luo et al., 2025). In research exploring type 2 diabetes mellitus (T2DM) treatment, BBP reduced blood glucose levels in T2DM rats, alleviated inflammation and lipid metabolism disorders, and mitigated tissue damage in the liver, spleen, kidneys, and pancreas. Mechanistic studies indicated that BBP reversed changes in blood and urine metabolites in T2DM rats. The key metabolic pathways involved were tryptophan metabolism, pentose and glucuronate interconversions, starch and sucrose metabolism, and glycerophospholipid metabolism. BBP also restored gut microbiota disorders in T2DM rats and increased short-chain fatty acid (SCFA) concentrations in the gut. These findings indicate that BBP improves T2DM by modulating multiple metabolic pathways, gut microbiota composition, and SCFA levels (Figure 2; Table 2) (Chen X. L. et al., 2022).

**TABLE 2:** Applications of bile-based TCMs in disease treatment.

Category of bile-based TCMs	Treated disease	Therapeutic effects and mechanisms
BBP	NASH	BBBP improves hepatic lipid and glucose metabolism by regulating the arginine biosynthesis pathway and activating the FXR/PXR-PI3K-AKT-NOS3 signaling pathway. It reduces fatty acid synthesis and promotes fatty acid oxidation (Jiang et al., 2023).
	HCC	BBP significantly reduces tumor volume and weight in a mouse model of HCC by inhibiting the STAT3 signaling pathway. It promotes apoptosis, suppresses cell proliferation, and inhibits tumor angiogenesis (Chen et al., 2020).
	Cholestatic liver injury	CBBP alleviates cholestatic liver injury by reducing the expression of inflammatory cytokines, suppressing the TLR4/MyD88/NF-κB signaling pathway, and downregulating apoptosis-related protein expression, thereby reducing hepatic inflammation (Cai et al., 2022).
	AMI	The combination of BBP and CF significantly reduces myocardial infarct size and pathological changes in rats with AMI. It also lowers serum levels of lactate dehydrogenase and creatine kinase, improving cardiac function (Luo et al., 2025).
	T2DM	BBP improves T2DM by modulating metabolic profiles, gut microbiota, and related metabolites (Chen X. L. et al., 2022).
PBP	MASLD	PBP inhibited excessive increases in body weight and liver weight in mice fed a high-fat diet. It alleviates dyslipidemia, reduces hepatic lipid accumulation and inflammation, and lowers the degree of liver injury (Kuang et al., 2023; Chen D. X. et al., 2022).
	DTH	PBP effectively reduces allergic responses in experimental models, such as bitter chloride-induced contact dermatitis and sheep red blood cell-induced paw swelling in mice (Kubo et al., 1989; Nakagami et al., 1990).
	UC	PBP significantly attenuates colonic inflammation in mice with UC. It improves mucosal structure, reduced inflammatory cell infiltration, and inhibits ulcer formation (He et al., 2011).
CBP	Neuroinflammation	Fermented cow bile significantly suppresses neuroinflammation in LPS-induced BV2 microglial cells. It inhibits the release of proinflammatory cytokines TNF-α and IL-1β, reduces NO and iNOS expression, and alleviates inflammation by suppressing NLRP3 inflammasome activation (Pei et al., 2022).

Although BBP has achieved notable therapeutic outcomes, the process of obtaining natural bear powder is extremely cruel to bears, not only causing long-term physical pain but also triggering serious ethical and legal controversies. For decades researchers have searched for substitutes that do not depend on endangered species yet provide equivalent therapeutic effects (Wang et al., 2012). The guiding principle is to match the chemical and pharmacological profile of bear bile. On this basis, four main categories are therefore considered: artificial bear bile, synthetic compounds, bile from other animals, and medicinal plants (Feng et al., 2009). Thus, bear bile substitutes already replicate the composition, efficacy and safety of natural bear bile, offering a scientifically sound and ethical means to end bear farming.

Artificial bear bile is a more complex substitute designed to replicate the holistic chemical fingerprint of natural bile. This product is typically manufactured using microbial biotransformation or enzymatic synthesis, where specific bacterial enzymes are used to catalyze the stereoselective epimerization of bile acids from other sources (Li et al., 2016; Yan et al., 2022). Unlike single synthetic compounds, artificial BBP aims to mimic the natural proportion of diverse taurine-conjugated bile acids, such as TUDCA and TCDCA. Analytical studies confirm that high-quality artificial BBP achieves over 95% similarity to natural bile in its chemical fingerprint, ensuring that the synergistic therapeutic effects of the original TCM formulations are preserved while offering superior stability and security (Li et al., 2016).

Synthetic compounds focus on the chemical manufacture of highly purified active ingredients found in natural bile, most notably UDCA and its taurine conjugate (TUDCA). UDCA was successfully synthesized by Japanese scientists as early as 1955 and has since become a cornerstone of Western medicine for dissolving gallstones and treating chronic cholestatic liver diseases (Kaplan, 1997; Rubin et al., 1994). These pure compounds offer significant advantages in clinical precision, as they allow for exact dosing and eliminate the variability associated with natural biological materials. While single-component synthetic drugs are effective for specific indications like PBC, they represent a shift from the multi-target, holistic approach of traditional medicine to a more targeted pharmacological model.

Bile from domestic animals, such as PBP and CBP, provides a widely available and cost-effective resource for substitution. Although their specific bile acid compositions vary, PBP is characterized by HDCA while CBP contains more GCA, they share fundamental pharmacological properties with bear bile, including significant anti-inflammatory, hepatoprotective, and sedative actions (Yan et al., 2022; Yan et al., 2017; Yan et al., 2016; Lin et al., 1997; Li et al., 1995). These animal-derived alternatives have been used in clinical practice for centuries and are documented in ancient texts like the *Compendium of Materia Medica*. Because they are by-products of the food industry, they offer a reliable and ethical medicinal source that avoids the ethical controversies and contamination risks associated with bear farming.

Medicinal plants represent an essential non-animal-derived pathway for the modernization of bile-based therapies. Traditional herbs such as *Coptis* (Huanglian) and *Scutellaria* (Huangqin) have been identified as potential herbal alternatives because they share similar therapeutic functions, such as clearing heat and detoxifying the liver (Feng et al., 2008). These plants contain bioactive alkaloids and flavonoids, such as berberine and baicalin, which have been shown to target the same inflammatory and metabolic signaling pathways influenced by bile acids. Plant-based substitutes are considered the most environmentally sustainable and ethically acceptable option, particularly as international regulations on animal-derived products become increasingly stringent. Integrating these plant-derived components into bile-based TCM research provides a scientifically sound path toward “Precision TCM” while ensuring the protection of endangered species.

### 3.2 PBP

The use of pig bile was first recorded in the ancient Chinese medical text *Ming Yi Bie Lu*, where it was described as “slightly cold in nature, capable of treating feverish illnesses and quenching heat-induced thirst” (Binghong and Jing, 2000). Later herbal classics also documented its properties (Lili et al., 1999). In the Ming Dynasty text *Compendium of Materia Medica* by Li Shizhen, pig bile is noted for its ability to “clear heat with its cold nature, relieve dryness with its lubricating property, and target the heart with its bitterness, while also removing heat from the liver and gallbladder”. Pig bile is described as having a bitter and slightly salty taste, a cold nature, and is affiliated with the liver, gallbladder, lung, and large intestine meridians. It is used to clear heat and dryness, relieve cough and asthma, and detoxify. Pig bile can be formulated into decoctions, pills, powders, and ointments, among other forms. Since its inclusion in the 2010 edition of the *Pharmacopoeia of the People’s Republic of China*, pig bile powder has been used to treat various conditions, including heat-related thirst, red eyes, jaundice, pertussis, asthma, dysentery, and abscesses (Chinese Pharmacopoeia Commission, 2010).

PBP has shown significant efficacy in the treatment of MASLD. In high-fat diet mouse models, PBP inhibited excessive weight gain and liver enlargement, improved lipid metabolism disorders, and reduced hepatic lipid accumulation and inflammation, thereby alleviating liver injury (Chen D. X. et al., 2022). Recent studies confirmed that PBP reduced MASLD phenotypes in mice fed a high-fat diet, including hepatic lipid droplet accumulation and liver weight. It also decreased serum alanine transaminase (ALT) levels (Kuang et al., 2023). The key component, HDCA, of PBP exerts therapeutic effects by inhibiting Ras-related nuclear protein (RAN)-mediated peroxisome proliferator-activated receptor alpha (PPARα) nuclear transport, enhancing PPARα nuclear localization, and activating PPARα-dependent fatty acid oxidation, thus alleviating MASLD (Kuang et al., 2023).

In addition, pig bile has been found to possess anti-delayed-type hypersensitivity (DTH) activity (Kubo et al., 1989; Nakagami et al., 1990). Studies showed that PBP significantly reduced allergic responses in mouse models of contact dermatitis induced by bitter chloride and ameliorated footpad swelling induced by sheep red blood cells. Its effect is mainly through immunomodulation during the sensitization phase of DTH, rather than through direct anti-inflammatory action (Kubo et al., 1989; Nakagami et al., 1990).

**FIGURE 3:**
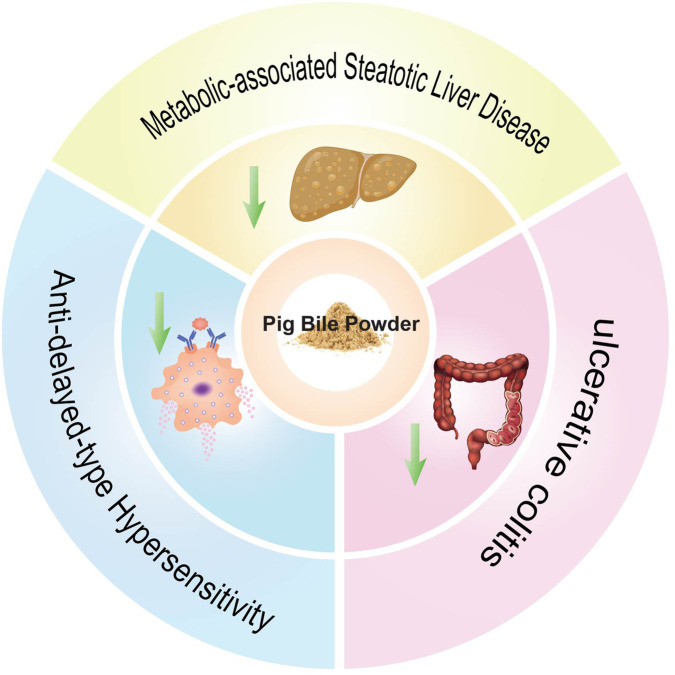
Clinical applications of PBP.

Furthermore, PBP reduced damage of colonic tissues in mice with ulcerative colitis (UC) (Zihua, 2020). It significantly improves trinitrobenzene sulfonic acid (TNBS)-induced colitis by improving mucosal structure, reducing inflammatory cell infiltration, and decreasing ulcer formation. Mechanistically, PBP inhibits myeloperoxidase (MPO) activity, reduces neutrophil infiltration, lowers cyclooxygenase-2 (COX-2) expression, and significantly decreases serum levels of tumor necrosis factor (TNF)-α and interleukin (IL)-6. These findings indicate that PBP has potential therapeutic value in the treatment of inflammatory bowel disease (Figure 3; Table 2) (He et al., 2011).

### 3.3 CBP

CBP has a long history of medicinal use, dating back to *Shennong’s Classic of Materia Medica*, with subsequent herbal texts also recording its applications. For example, *Mingyi Bie Lu* highlights that it “eliminates internal heat, quenches thirst, relieves dry mouth, and benefits the eyes”. Meanwhile, *Compendium of Materia Medica* states that it can “reduce jaundice, kill parasites, and treat swellings and abscesses”. These texts collectively underscore the extensive application of cow bile in traditional Chinese medicine (Yan et al., 2016).

As an animal-derived medicine, CBP has anti-inflammatory, antipyretic, and choleretic effects. It also has sedative or anesthetic effects on the central nervous system, respiratory system, and circulatory system. It has been used to treat indigestion and constipation (Pei et al., 2022). In clinical practice, CBP is used to treat pharyngitis, upper respiratory tract infections, dyspepsia, and icteric infectious hepatitis, exhibiting a wide range of therapeutic applications (Xinlei, 1998). Studies have found that fermented cow bile significantly enhanced anti-inflammatory effects by inhibiting the NLRP3 inflammasome. UHPLC-MS/MS analysis showed that cow bile suppressed release of the pro-inflammatory cytokines TNF-α and IL-1β in lipopolysaccharide (LPS)-induced BV2 microglial cells. It also reduced the expression of nitric oxide (NO) and inducible nitric oxide synthase (iNOS). Moreover, it inhibited neuroinflammation by attenuating activation of the NLRP3 inflammasome (Pei et al., 2022). These findings suggest new directions and strategies for the application of bovine bile powder (Table 2).

## 4 The roles of major components of bile-based TCMs in the treatment of liver diseases

### 4.1 HDCA

HDCA, a major component of PBP, plays a key role in the treatment of various liver diseases. Its properties strongly support the clinical use of pig bile powder. HDCA has shown significant therapeutic effects in MASLD. Specifically, HDCA directly interacts with the RAN protein and inhibits formation of the RAN/CRM1/PPARα heterotrimer complex, which mediates nucleocytoplasmic shuttling. This inhibition promotes nuclear accumulation and localization of PPARα, thereby activating PPARα-dependent fatty acid oxidation and relieving MASLD symptoms. Moreover, HDCA has been shown to ameliorate insulin resistance and liver inflammation, suggesting its potential benefits in treatment of metabolic disorders (Zhong et al., 2023). Recent studies have found that serum HDCA levels are significantly reduced in MASLD patients and are negatively correlated with disease severity. Animal studies have confirmed that HDCA can improve MASLD by regulating the gut–liver axis. On the one hand, HDCA suppresses the intestinal FXR signaling pathway and reduces FGF15 production. On the other hand, HDCA promotes the growth of beneficial gut bacteria, such as *Parabacteroides distasonis*. These bacteria produce γ-linolenic acid, which activates hepatic PPARα and subsequently increases expression of FXR and CYP7B1 in the liver. In the gut-liver axis, after *P. distasonis* is enriched by HDCA, the bacterium uses lipid substrates from the high-fat diet to synthesise γ-linolenic acid (C18:3). This metabolite reaches the liver through the portal vein, binds to PPARα, and activates the PPARα pathway. Activated PPARα suppresses CYP7A1 transcription, blocks the classic bile-acid synthetic route, and shifts synthesis to the alternative CYP7B1-mediated pathway. The result is less hepatic lipid accumulation and reduced inflammation. Furthermore, HDCA has shown superior efficacy compared with current drugs such as obeticholic acid (OCA) or UDCA, indicating its potential as a novel therapeutic strategy for MASLD (Kuang et al., 2023).

Emerging evidence also suggests that HDCA has a protective effect against cholestatic hepatic fibrosis (CHF). HDCA promotes expression of ETV4, increases secretion of MMP9, and enhances extracellular matrix (ECM) degradation, thereby reducing liver fibrosis. Mechanistically, HDCA facilitates m6A modification of ETV4 mRNA, which improves its translational efficiency. This modification recruits IGF2BP1 and PABPC1, inhibiting deadenylation of ETV4 mRNA, enhancing mRNA stability, prolonging its storage in P-bodies, and promoting translation. These findings highlight ETV4 as a key antifibrotic target of HDCA in the treatment of cholestatic hepatic fibrosis (Table 3) (Xue et al., 2025).

**TABLE 3:** Therapeutic effects of major components of bile-based TCMs on liver diseases.

Major component of bile-based TCMs	Treated disease	Therapeutic effects and mechanisms
HDCA (major component of PBP)	MASLD	HDCA alleviates MASLD symptoms by inhibiting RAN-mediated nuclear-cytoplasmic transport of PPARα, enhancing its nuclear localization, and activating PPARα-dependent fatty acid oxidation. HDCA also improves MASLD in mice by acting through the gut-liver axis. It suppresses intestinal FXR signaling and enriches beneficial gut microbiota, thereby increasing hepatic expression of CYP7B1, PPARα, and FXR (Kuang et al., 2023).
	CHF	HDCA induces ETV4 expression in cholangiocytes, which promotes the secretion of MMP9. This enhances extracellular matrix degradation and reduces cholestatic liver fibrosis (Xue et al., 2025).
UDCA (major component of BBP)	PBC	UDCA decreases serum levels of ALP, AST, ALT, and TBIL in patients with PBC (Zhang et al., 2013).
	PSC	UDCA reduces serum levels of AST and ALP in patients with PSC (Lindor et al., 2009).
	NASH	UDCA treatment significantly improves liver function in patients with NASH, as shown by reduced ALT and γ-GT levels, and improved liver fibrosis (Xiang et al., 2013).
TUDCA (major component of BBP)	HS-induced liver injury	TUDCA shows potential therapeutic effects against HS-induced liver injury by regulating the SIRT1-FXR signaling pathway and its downstream targets (Sun et al., 2020).
	Liver cirrhosis	TUDCA significantly decreases serum ALT, AST, and ALP levels in patients with liver cirrhosis, while increasing serum albumin. Its mechanisms may include promoting bile secretion, reducing bile acid toxicity, inhibiting apoptosis, and relieving ER stress (Pan et al., 2013).
	MASLD	TUDCA improves intestinal inflammation and barrier function, reduces intestinal fat transport, and modulates gut microbiota composition to alleviate HFD-induced MASLD in mice (Wang et al., 2018). In addition, TUDCA reduces hepatic fat accumulation by regulating gut microbiota and bile acid metabolism (Wang et al., 2024).
	IRI-induced bile duct injury and cholestasis	TUDCA reduces bile duct injury and cholestasis in rats with IRI by modulating the PKCα–ezrin signaling pathway (Baiocchi et al., 2008).
	HCV infection	TUDCA inhibits HCV infection by suppressing ER stress and its downstream signaling pathways (Lin et al., 2023).

### 4.2 UDCA

UDCA, a major component of BBP, is the first FDA-approved drug for primary biliary cirrhosis (PBC). PBC is an autoimmune disease characterized by progressive inflammation and destruction of intrahepatic bile ducts, eventually leading to cirrhosis. Studies have shown that UDCA reduces serum levels of alkaline phosphatase (ALP), aspartate aminotransferase (AST), ALT, and total bilirubin (TBIL) in patients with PBC (Zhang et al., 2013). While UDCA effectively reduces serum biochemical indicators, its impact on mortality and liver transplant rates is not significant (Ishibashi et al., 2011). Moreover, about 40% of PBC patients have an incomplete response to UDCA, meaning that significant biochemical improvement is not achieved (Lammers et al., 2014). New therapies, such as UDCA combined with budesonide or fenofibrate, are under clinical study (Dyson et al., 2015; Czul and Levy, 2016). However, Lindor et al. reported that high-dose UDCA (28–30 mg·kg^−1^·d^−1^ for 5 years) increased the risk of advanced cirrhosis, esophageal varices, and cholangiocarcinoma (Lindor et al., 2009). A phase-IV-trial led by West China Hospital of Sichuan University (NCT03345589) tested an intermediate dose of 18–22 mg·kg^−1^·d^−1^ in refractory PBC. The results showed that the standard dose of UDCA (13–15 mg·kg^−1^·d^−1^) achieved biochemical responses in approximately 60%–70% of PBC patients. After dose escalation to 18–22 mg·kg^−1^·d^−1^ for 6 months, the response rate rose from 36% to 59%, UK-PBC risk score and fibrosis index both fell, and adverse events remained mild (Xiang et al., 2021).

Primary sclerosing cholangitis (PSC) is a chronic cholestatic liver disease of unknown cause, characterized by inflammation and fibrosis of the bile ducts, leading to bile stasis and liver cirrhosis. In a randomized, double-blind, controlled trial, patients receiving UDCA exhibited greater reductions in AST and ALP levels compared with the placebo group (Lindor et al., 2009). However, several randomized controlled trials and meta-analyses have reported that UDCA does not significantly lower mortality, liver transplant risk, or the incidence of cholangiocarcinoma and colorectal cancer in PSC patients (Othman et al., 2012; Hansen et al., 2013). Nevertheless, some studies have shown that UDCA treatment is significantly associated with improved pre-liver transplant survival in PSC patients (Arizumi et al., 2022). Consequently, UDCA may benefit certain PSC patients, but further research is needed to determine its optimal use and dosage.

The efficacy of UDCA in the treatment of NASH varies. A systematic review that included seven randomized controlled trials with a total of 655 participants found that UDCA significantly lowered ALT, AST, and γ-GT (γ-glutamyltransferase) in MASLD patients, but had no significant effect on ALP or bilirubin levels (Patel et al., 2024). For high-dose UDCA, some studies have reported significant improvements in ALT, γ-GT, and liver fibrosis, while others have found no meaningful changes (Xiang et al., 2013; Leuschner et al., 2010). In NASH mouse models, 300 mg/kg/day UDCA improved liver inflammation and modulated gut microbiota and bile acid metabolism (Li et al., 2024). UDCA has potential for NASH treatment, but there is individual variation and more research is needed to confirm its efficacy.

### 4.3 TUDCA

TUDCA accounts for approximately 40% of BBP and is one of its main components. TUDCA plays an important role in the therapeutic effects of BBP and has shown promising results in the treatment of various liver disorders. Studies have demonstrated that TUDCA exerts protective effects in hemorrhagic shock (HS)-induced liver injury. In HS rat models, TUDCA significantly reduced liver inflammation and apoptosis, promoted hepatocyte proliferation, and alleviated HS-induced liver damage. Mechanistically, TUDCA upregulated SIRT1-FXR activity, suppressing NF-κB and p53 expression and acetylation, and increased FoxM1 expression. These findings suggest that TUDCA may have therapeutic potential against HS-induced liver injury through modulation of the SIRT1-FXR pathway (Sun et al., 2020).

TUDCA has also been shown to have potential efficacy in liver cirrhosis and was well-tolerated. A randomized, double-blind controlled trial showed that after 6 months treatment, patients receiving TUDCA exhibited significant reductions in serum ALT, AST, and ALP levels (42.8%, 45.9%, and 38.4% from baseline) and a 16.8% increase in albumin levels. In the UDCA group, AST and albumin levels were also improved but to a lesser extent than in the TUDCA group. The therapeutic mechanism of TUDCA in liver cirrhosis may involve promotion of bile secretion, reduction of endogenous bile acid toxicity, inhibition of apoptosis, and alleviation of endoplasmic reticulum stress (Pan et al., 2013).

Significant therapeutic effects of TUDCA have been observed in MASLD mouse models. TUDCA mitigated high-fat diet (HFD)-induced MASLD progression by improving gut inflammation and barrier function, decreasing fat transport, and modulating gut microbiota composition (Wang et al., 2018). Another study showed that TUDCA raises the abundance of bile-salt-hydrolase (BSH)-producing bacteria, particularly *Bifidobacterium*. Higher BSH activity increases unconjugated bile acids, suppresses intestinal FXR-FGF15 signaling, relieves the inhibition of hepatic CYP7A1, and promotes cholesterol conversion to bile acids. Concurrently, TUDCA downregulates hepatic FATP5, lowers fatty-acid uptake, and upregulates NTCP to accelerate bile-acid recycling. These combined effects reduce hepatic lipid content through a “microbiota-metabolite-receptor” axis and thereby counteract high-fat-diet-induced steatosis (Wang et al., 2024).

TUDCA protects against liver injury induced by hepatic ischemia-reperfusion injury (IRI). It lowered hepatocyte cholesterol accumulation, improved liver function, and reduced inflammation and apoptosis. By modulating ezrin expression and distribution, TUDCA maintained bile duct morphology and function, protected microvillus structures, and decreased cholestasis and duct injury. It also protected microvillus structures by enhancing PKCα expression and its translocation to the membrane, thereby reducing cholestasis and duct injury (Baiocchi et al., 2008). These findings suggest that TUDCA alleviates IRI-induced liver injury through the PKCα-ezrin pathway.

As an endoplasmic reticulum (ER) stress inhibitor, TUDCA has been shown to suppress chronic hepatitis C virus (HCV) infection and HCV replicons. It alleviated ER stress by reducing PERK and ATF6α signaling, reduced phosphorylation of PKA substrates and GSK-3β at serine 9, and decreased β-catenin levels. This inhibited Wnt/β-catenin pathway activation. Moreover, TUDCA demonstrated antiviral effects by significantly inhibiting extracellular HCV infection in a dose-dependent manner. Thus, TUDCA provides a potential novel strategy for HCV treatment by suppressing ER stress and downstream signaling pathways (Lin et al., 2023).

### 4.4 Bile acid-mediated modulation of the gut-liver axis

The therapeutic potential of bile-based TCMs is realized through a reciprocal feedback loop within the gut-liver axis. This process involves the remodeling of microbial ecology, the reinforcement of the epithelial barrier, and the subsequent reprogramming of hepatic metabolic signaling.

#### 4.4.1 Microbiota remodeling and ligand diversity

Bile acids from BBP and PBP act as selective chemical pressures that reshape the intestinal microenvironment. This modulation is not merely a change in bacterial abundance but a fundamental shift in the metabolic capacity of the microbiota. For instance, TUDCA from BBP raises the abundance of *Bifidobacterium*, which possesses high BSH activity (Wang et al., 2024). These microbial enzymes catalyze the deconjugation of bile acids, effectively altering the hydrophobicity and signaling potency of the total bile acid pool. This shift transforms the ligand profile available for intestinal FXR and TGR5, thereby determining the strength of the feedback signals sent to the liver. Such “microbiota-mediated ligand diversification” explains how bile-based TCMs can exert systemic effects far beyond their initial chemical forms.

#### 4.4.2 Reinforcement of physical and immunological barriers

The intestinal barrier serves as the first line of defense against liver inflammation. The depth of protection offered by bile-based TCMs, particularly TUDCA, lies in its role as a chemical chaperone that suppresses ER stress within intestinal epithelial cells (Wang et al., 2018). By inhibiting the PERK and ATF6α pathways, TUDCA prevents the inflammatory disassembly of tight junction proteins (such as occludin and ZO-1). Furthermore, bile acids modulate the intestinal immunological barrier by suppressing the activation of the NLRP3 inflammasome in gut-associated lymphoid tissues. This dual reinforcement prevents the translocation of LPS and other PAMPs into the portal vein, which is a prerequisite for mitigating chronic hepatic inflammation and the subsequent activation of Kupffer cells.

#### 4.4.3 Remote hepatic reprogramming via gut-derived signals

The final stage of this axis involves the remote regulation of hepatic transcription by gut-derived molecular messengers. A primary mechanism is the gut-restricted FXR-FGF15/19 axis. Activation of intestinal FXR by TCM-derived ligands triggers the secretion of fibroblast growth factor 15/19 (FGF15/19), which travels through the portal circulation to bind with FGFR4 in the liver. This signal specifically suppresses the transcription of CYP7A1, the rate-limiting enzyme in classical bile acid synthesis, thereby preventing bile acid overload and hepatotoxicity (Kuang et al., 2023; Wang et al., 2024). Additionally, microbial metabolites induced by HDCA, such as γ-linolenic acid, act as remote ligands for hepatic PPARα, shifting the liver from lipogenesis to fatty acid oxidation (Kuang et al., 2023). By synthesizing these mechanisms, it is evident that bile-based TCMs function as systemic regulators that restore homeostatic balance across the entire gut-liver landscape through multi-step molecular signaling.

## 5 Abnormally elevated bile acid components are positively correlated with the progression and severity of liver diseases

### 5.1 TCA

TCA is not only a component of CBP and BBP but also a major endogenous conjugated bile acid in humans. Studies have found elevated levels of TCA in the serum of patients with drug-induced liver injury (DILI) compared with healthy controls. TCA levels increase progressively with elevated bilirubin and disease severity. Additionally, changes in TCA levels are correlated with bilirubin levels in patients with chronic hepatitis B (CHB), suggesting that TCA may contribute to liver injury beyond DILI. A reduction in hepatic expression of the bile salt export pump (BSEP) is associated with elevated TCA levels. This suggests that increased levels of TCA may result from impaired bile acid excretion in the liver. Therefore, TCA is not only a biomarker that is closely correlated with the severity of drug-induced liver injury (DILI) but may also play a role in predicting clinical outcomes in DILI (Tian et al., 2021).

Moreover, TCA has been shown to have significant immunosuppressive effects in HBeAg-positive CHB patients. Research has indicated that serum bile acid levels, especially TCA, are markedly elevated in these patients compared to healthy controls and other HBV infection stages. High concentrations of TCA impair the effector functions of CD3^+^CD8^+^ T cells and natural killer (NK) cells, leading to reduced cell numbers and frequencies. TCA also decreases production of cytotoxic granules (such as granzyme B and perforin) and cytokines (such as interferon (IFN)-γ and TNF-α). Additionally, TCA suppresses the immunomodulatory activity of IFN-α, weakening the therapeutic effect of IFN-α treatment. Thus, TCA impairs the ability of the immune system to clear HBV by suppressing CD8^+^ T cell and NK cell functions, which contributes to disease progression. These findings suggest that targeting TCA may be a promising strategy to restore IFN-α responsiveness in CHB patients (Table 4) (Xun et al., 2021).

**TABLE 4:** Positive correlation between abnormally elevated bile acid components and the severity of liver disease progression.

Major component of bile-based TCMs	Disease	Function and mechanism
TCA (major component of BBP and CBP)	DILI	Serum levels of TCA are significantly higher in patients with DILI compared to healthy controls. TCA levels increase with rising bilirubin concentrations and disease severity. TCA is a biomarker closely associated with the severity of DILI (Tian et al., 2021).
	CHB	TCA suppresses the effector function of CD8^+^ T cells and NK cells, which reduces the immune system’s ability to eliminate HBV. This suppression contributes to the progression of CHB (Xun et al., 2021).
GDCA (major component of PBP)	CFLD	Patients with CF show higher serum levels of GDCA compared to healthy individuals. Changes in GDCA levels are related to the severity of CFTR genotype, GGTP activity, and pancreatic insufficiency. These factors affect bile acid metabolism and liver disease development. GDCA is a potential biomarker for CF-related liver disease (Drzymala-Czyz et al., 2022).
GCA (major component of CBP)	Cholestatic liver fibrosis	GCA promotes YAP nuclear translocation and CTGF expression, which activates fibrogenic pathways in cholestatic liver fibrosis. This process contributes to disease progression. GCA is a potential biomarker for cholestatic liver fibrosis (Yuan et al., 2023).

### 5.2 GDCA

GDCA is one of the major components of PBP. It is also an endogenous conjugated bile acid in humans. Studies have shown that serum bile acid levels are generally higher in patients with cystic fibrosis (CF) compared with healthy individuals. Among these, GDCA levels are significantly elevated in CF patients with liver disease, but not in those without liver involvement. This suggests a potential link between GDCA and the progression of cystic fibrosis-related liver disease (CFLD). Changes in GDCA levels are also associated with the severity of cystic fibrosis transmembrane conductance regulator (CFTR) gene mutations, γ-Glutamyltransferase activity, and pancreatic insufficiency. These factors together influence bile acid metabolism and the development of liver disease. Therefore, GDCA may serve as a potential biomarker for CFLD and could play an important role in monitoring disease progression (Table 4) (Drzymala-Czyz et al., 2022).

### 5.3 GCA

GCA is the most abundant component of CBP. It is also an endogenous conjugated bile acid in humans. Studies have shown that GCA levels are abnormally elevated in the serum of patients with cholestatic liver diseases. In in vitro experiments, GCA significantly promoted secretion of connective tissue growth factor (CTGF) in mouse and rat primary hepatocytes, as well as in HepaRG cells, compared with other bile acids. GCA increases CTGF expression by promoting nuclear translocation of Yes-associated protein (YAP), which activates hepatic stellate cells and contributes to liver fibrosis. In a bile duct ligation mouse model, GCA administration further aggravated liver fibrosis and increased hepatic CTGF expression. Therefore, GCA exerts a profibrotic effect by promoting YAP nuclear translocation and CTGF expression, which accelerates progression of cholestatic liver fibrosis (Table 4) (Yuan et al., 2023).

## 6 Dual effects of bile-based TCMs in the treatment of liver diseases

Liver disease is a global health challenge with complex pathological factors. Bile-based TCMs show great potential for treatment, but the opposing actions of their internal components create challenges for clinical application. To improve efficacy, it is necessary to evaluate the interactions between these components based on the “Yin-Yang” balance theory.

### 6.1 Quantitative pharmacological basis of the Yin-Yang framework

The “Yin-Yang” balance of bile-based TCMs is mechanistically defined by the ratio of hydrophilic to hydrophobic bile acids and their specific receptor activation parameters. The “Yang” (beneficial) effect is driven by hydrophilic components like UDCA and TUDCA, which possess low hydrophobicity (Heuman index of −0.47 for UDCA) (Heuman, 1989). UDCA is a weak ligand for FXR with an EC_50_ exceeding 500 μM, ensuring it does not trigger intrahepatic toxicity at therapeutic doses (Parks et al., 1999). In contrast, the “Yin” (disease-promoting) effect is characterized by more hydrophobic bile acids such as TCA, GDCA, and GCA, which have higher hydrophobicity indices (+0.25 to +0.54) and distinct pathological roles (Heuman, 1989). TCA is a potent FXR ligand (EC_50_ ≈ 10–15 μM), but at elevated serum levels, it suppresses immune effector cells (CD8^+^ T and NK cells), facilitating viral persistence in chronic hepatitis B (Xun et al., 2021; Parks et al., 1999). GCA and GDCA are significantly elevated in cholestatic and cystic fibrosis-related liver diseases, where they serve as biomarkers of progression (Drzymala-Czyz et al., 2022). Specifically, GCA promotes liver fibrosis by activating YAP and CTGF signaling when its concentration exceeds normal homeostatic levels (Yuan et al., 2023). While total serum bile acids in healthy individuals remain below 10 μM, the accumulation of “Yin” components like TCA, GDCA, or GCA beyond a threshold of 50–100 μM triggers mitochondrial dysfunction and hepatocyte apoptosis (Tian et al., 2021; Attili et al., 1986). This shift in bile acid pool dynamics, from balanced signaling to a “Yin” dominant state characterized by high concentrations of hydrophobic conjugates, provides a measurable pharmacological basis for the progression and severity of liver diseases.

### 6.2 Dual effects of BBP in treating liver diseases

**FIGURE 4:**
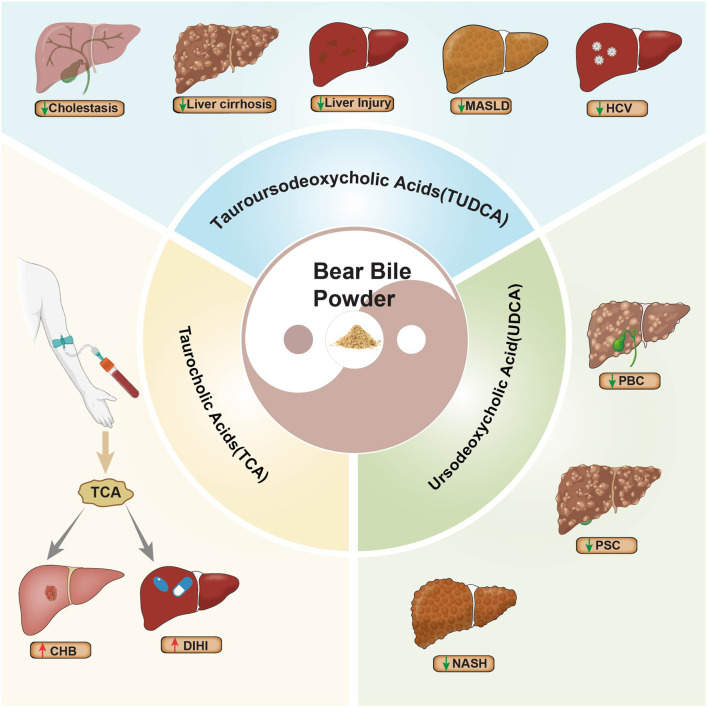
The dual effects of BBP in treating liver diseases.

In BBP, TUDCA and UDCA are the primary “Yang” components that provide significant therapeutic benefits. However, BBP also contains TCA, which may act as a disease-promoting factor. Research shows that elevated serum TCA is positively correlated with the severity of DILI (Tian et al., 2021). In patients with HBeAg-positive CHB, TCA demonstrates immunosuppressive effects (Xun et al., 2021) (Figure 4). Because BBP contains both therapeutic (TUDCA/UDCA) and potentially harmful (TCA) bile acids, its clinical utility may be limited if these components are not properly balanced.

### 6.3 Dual effects of PBP in treating liver diseases

**FIGURE 5:**
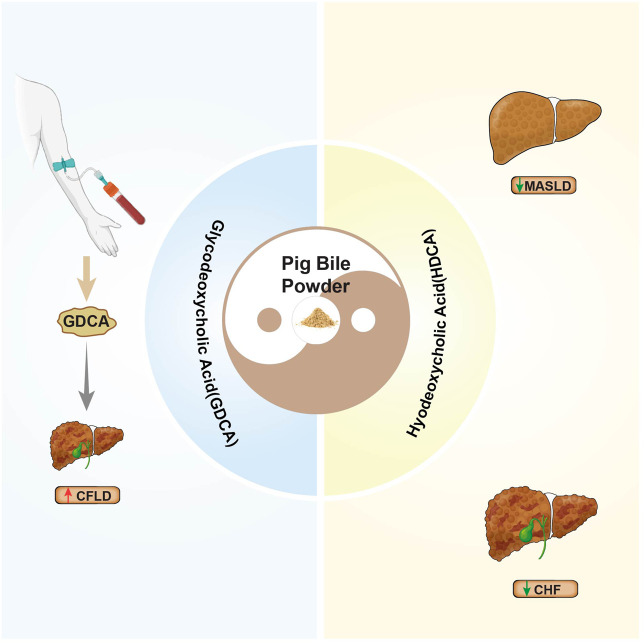
The dual effects of PBP in treating liver diseases.

Similarly, PBP exhibits dual effects through its main components. HDCA is a protective “Yang” factor that improves symptoms of MASLD and cholestatic CHF by activating PPARα and inhibiting fatty acid oxidation (Kuang et al., 2023; Zhong et al., 2023; Xue et al., 2025). Conversely, GDCA serves as a “Yin” factor. High levels of endogenous GDCA are closely linked to the progression of CFLD (Drzymala-Czyz et al., 2022) (Figure 5). The presence of GDCA in PBP may counteract the therapeutic benefits of HDCA, which limits the application of PBP in certain liver conditions.

### 6.4 Receptor-level counterbalance: FXR and TGR5

The opposing actions of bile acids are also reflected in their primary receptors, FXR and TGR5, through competitive binding and differential activation mechanisms. In the complex environment of the bile acid pool, different bile acid species serve as ligands with varying binding affinities and transcriptional activities for the same receptor. For instance, while potent agonists like CDCA strongly activate FXR to regulate lipid metabolism, other components in bile-based TCMs may act as weak agonists or even functional antagonists depending on the cellular milieu (Kim and Fang, 2018). The overall effect of the bile acid pool on liver homeostasis is determined by the collective signaling output of these diverse ligands. When the “Yang” components dominate, they promote protective gene expression; however, when the receptor-level balance is disrupted by the over-accumulation of “Yin” components, it may trigger pathological pathways. This synergistic “Yin-Yang” mechanism is evident in glucose regulation, where gut-restricted FXR activation induces TGR5 expression, amplifying cAMP signaling to improve insulin sensitivity (Kim and Fang, 2018). In viral infections, the host defense relies on this dynamic equilibrium: TGR5 activation initiates innate antiviral immunity via the TGR5-β-arrestin-SRC axis (Hu et al., 2019), whereas FXR activation may suppress type-I interferon expression, facilitating viral persistence (Liang et al., 2024). This intricate receptor-level antagonism ensures that the liver can respond flexibly to metabolic and inflammatory stresses (Table 5).

**TABLE 5:** Comparison of FXR and TGR5 functions and pathological implications.

Feature	FXR	TGR5
Tissue distribution	Liver, intestine, kidney, adrenal glands (Mudaliar et al., 2013)	Enteroendocrine cells, gallbladder, immune cells, muscle (Zheng et al., 2021)
Metabolic effects	Inhibits bile acid synthesis; regulates glucose/lipid metabolism (Kim and Fang, 2018)	Stimulates GLP-1 secretion; improves insulin sensitivity (Zheng et al., 2015)
Immunological roles	Mostly anti-inflammatory; suppresses IRF3/IFN signaling (Liang et al., 2024)	Pro-inflammatory for antiviral defense; enhances IFN via SRC (Hu et al., 2019)
Fibrosis modulation	Anti-fibrotic (inhibits HSC activation) (Xue et al., 2025)	Complex; may reduce inflammation-driven fibrosis (Li et al., 2011)
Viral implications	May facilitate viral persistence by inhibiting IFN (Liang et al., 2024)	Promotes viral clearance via TGR5-SRC-IFN axis (Hu et al., 2019)
Adverse effects	Dyslipidemia (increased LDL-C), pruritus, hepatotoxicity (Nevens et al., 2016)	Gallbladder enlargement, itch, potential IFN-induced cell death (Li et al., 2011; Alemi et al., 2013)
Beneficial condition	NASH, cholestasis, obesity (Jiang et al., 2023; Fang et al., 2015)	Type 2 diabetes, acute viral infection (Zhang et al., 2025; Lun et al., 2024)
Detrimental condition	Early-stage viral infection (prevents clearance) (Liang et al., 2024)	Chronic over-activation (gallbladder stasis/cell death) (Briere et al., 2015)

#### 6.4.1 Safety profile and risk-benefit considerations

While FXR and TGR5 agonists offer therapeutic benefits, their activation is not without risks. A careful risk-benefit analysis is crucial, especially when considering dose-dependent effects and potential long-term complications. Systemic FXR activation, particularly by potent synthetic agonists, can cause liver toxicity. For example, clinical trials with OCA, an FXR agonist, have shown dose-dependent hepatotoxicity. At higher doses (e.g., 25 mg/day), patients exhibited increased serum ALP and ALT/AST levels (Nevens et al., 2016). This occurs because strong FXR activation can sometimes lead to an overload of bile acids in the liver. FXR normally helps manage bile acid levels. However, if this system is over-stimulated, it can reduce the expression of BSEP, which is responsible for removing bile acids from liver cells (Tian et al., 2021). This impaired removal causes bile acids, particularly hydrophobic ones, to accumulate inside hepatocytes. This intracellular bile acid accumulation directly triggers stress, inflammation, and can lead to liver cell death (Tian et al., 2021). Specifically, high serum levels of TCA are positively correlated with the severity of DILI in patients (Tian et al., 2021). Similarly, elevated GCA promotes liver fibrosis by activating profibrotic pathways (Yuan et al., 2023). This means that while some bile acids are therapeutic, their excessive accumulation can become toxic.

The bile acid receptors FXR and TGR5 generally function as tumor suppressors and cell protectors in the liver. FXR maintains metabolic homeostasis and suppresses hepatocarcinogenesis by inhibiting hepatic inflammation and promoting DNA repair (Huang et al., 2015; Huang et al., 2022). Studies show that FXR expression is significantly reduced in human hepatocellular carcinoma (HCC) tissues, often due to downregulation by pro-inflammatory cytokines like TNF-α and IL-6 (Su et al., 2012). However, the role of FXR in cancer remains complex and somewhat controversial; while its absence leads to spontaneous liver tumors in mice, some research suggests that FXR activation might stimulate cell proliferation in specific human HCC cell lines (Fujino et al., 2012; Kumagai et al., 2013). Similarly, TGR5 is considered a liver tumor suppressor, and its promoter is frequently hypermethylated in HCC patients, leading to reduced receptor activity (Han et al., 2014; Wang et al., 2013). Although TGR5 activation typically inhibits the proliferation of liver cancer cells, potential tumorigenic risks exist with chronic activation in specific contexts (Han et al., 2014). For instance, TGR5 is often overexpressed in cholangiocarcinoma and cystic bile duct cells, where it may promote cell survival and cyst growth (Ma et al., 2021; Keitel and Haussinger, 2018). Furthermore, the FXR-mediated induction of FGF19 has been associated with pro-tumorigenic actions in the liver (Huang et al., 2022; Piglionica et al., 2018). Therefore, while both receptors offer hepatoprotection, their therapeutic application requires careful monitoring to balance their protective effects against potential risks of promoting cell proliferation in different tissue types (Huang et al., 2015; Ma et al., 2021).

TGR5 activation directly affects gallbladder function, which has significant clinical safety implications. TGR5 is highly expressed in the gallbladder epithelium (Li et al., 2011). When TGR5 is activated by agonists (e.g., INT-777 or LCA), it stimulates gallbladder filling by relaxing the smooth muscle and promoting epithelial secretion (Li et al., 2011). In clinical trials, this effect has led to side effects such as gallbladder enlargement and bile stasis (Briere et al., 2015). For example, studies with TGR5 agonists showed instances of gallbladder dilation (Briere et al., 2015). While such effects may not immediately cause gallstones, chronic bile stasis can increase the risk of stone formation and cholecystitis over time (Li et al., 2011). This means that systemic TGR5 activation can lead to discomfort and potential complications related to gallbladder health, impacting patient safety and limiting the broad clinical use of systemic TGR5 agonists. This risk emphasizes the need for developing tissue-specific TGR5 agonists that target beneficial pathways without affecting the gallbladder (Zhang et al., 2025).

### 6.5 Clinical application and pathological transformation

The clinical application of bile-based TCMs requires a deep understanding of “Yin-Yang transformation,” where the role of specific bile acids may change during different stages of disease progression. In the acute phase of liver disease, characterized by intense inflammation or “Liver Fire” in TCM theory, the primary goal is to provide rapid hepatoprotection and anti-inflammatory effects. During this stage, increasing the proportion of therapeutic “Yang” components (such as TUDCA or HDCA) is critical to suppress ER stress and prevent massive hepatocyte apoptosis (Cai et al., 2022; Sun et al., 2020). However, as the disease transitions into a chronic or remission phase, the therapeutic focus must shift. In these smoldering stages, the accumulation of “Yin” components (such as GCA or TCA) becomes the core driver of pathology, as they can persistently activate fibrogenic pathways or cause chronic immune suppression (Xun et al., 2021; Yuan et al., 2023). Therefore, the dosage and composition of bile-based TCMs must be dynamically adjusted to prevent the pathological transformation of the bile acid pool. For example, reducing the accumulation of pro-fibrotic GCA is essential to prevent the progression from chronic inflammation to cirrhosis. This dynamic treatment strategy ensures that bile-based TCMs maintain a “Yin-Yang balance” in the human body, providing a scientific basis for the precision application of these traditional medicines in modern hepatology.

## 7 Functional differences of bile acids across tissues limit the clinical application of bile-based TCMs

The pharmacological activities of bile-based TCMs, as detailed in the previous component-specific analyses, are fundamentally determined by how their constituent bile acids act as ligands for the FXR and TGR5 receptors. The “Yin-Yang” balance described in bile-based TCMs is not merely a conceptual framework but reflects the net outcome of complex ligand-receptor interactions across different organs. While “Yang” components like TUDCA and HDCA promote protective signaling, “Yin” components like TCA and GCA can trigger pathological responses if their concentrations exceed safety thresholds. The clinical challenge arises because these receptors are expressed in multiple tissues, including the liver, intestine, and gallbladder, and their activation can produce vastly different biological effects depending on the site. Therefore, understanding the tissue-specific functional differences of FXR and TGR5 is essential to explain why the same bile acid component can be therapeutic in one context but toxic in another.

The widespread expression and functional diversity of FXR in multiple tissues, including the liver, intestine, skin, and immune system, tightly intertwine its therapeutic efficacy with tissue-specific toxic side effects, which constitutes the main limitation of its clinical transformation. Phase III clinical studies have confirmed that OCA (a potent FXR agonist) significantly improves biochemical markers in PBC patients. However, OCA exhibits dose-dependent hepatotoxicity, manifested by increased serum alkaline phosphatase and transaminase levels (Nevens et al., 2016). The intestine is a key organ for FXR function and also an important source of systemic side effects. Additionally, Phase II studies have showed that OCA improves insulin resistance, hepatic inflammation, and fibrosis in patients with MASLD. Nevertheless, in the high-dose OCA group (25 mg/day), significantly increases in total cholesterol and low-density lipoprotein cholesterol (LDL-C) were observed, along with markedly higher incidence of pruritus (Mudaliar et al., 2013). These clinical findings for OCA provide a benchmark for evaluating the “Yin” components of bile-based TCMs. Although bile-based TCMs are multi-component and exert milder effects than synthetic agonists, their core ligands, such as TCA and GCA, have the potential to trigger similar hepatotoxic responses if they accumulate excessively and over-activate intrahepatic FXR signaling. Intestine-specific FXR activation can produce markedly different metabolic effects compared to systemic FXR activation. Systemic FXR agonists such as OCA may cause dyslipidemia and pruritus, while selective intestinal FXR activators like Fexaramine can avoid direct hepatic activation, circumvent related toxicity risks, and significantly improve obesity and insulin resistance in animal models (Fang et al., 2015).

TGR5 is mainly expressed in enteroendocrine L cells of the distal ileum and colon and can be activated by bile acid agonists, including natural bile acid analogs (such as DCA) and synthetic small-molecule agonists (such as INT-777). TGR5 activation promotes glucagon-like peptide-1 (GLP-1) secretion, which subsequently influences insulin secretion and glucose utilization, making it a potential therapeutic target for type 2 diabetes (Zheng et al., 2015; Zheng et al., 2021; Lun et al., 2024). However, due to its side effects including hepatotoxicity, gallbladder enlargement, pro-inflammatory properties, and potential carcinogenicity, the clinical treatment of TGR5 agonists faces severe challenges (Li et al., 2011; Briere et al., 2015; Hunt et al., 2020; Alemi et al., 2013; Cao et al., 2016). For example, TGR5 agonists (INT-777, LCA) trigger dual pathophysiological effects through the cAMP-PKA signaling pathway: they relax smooth muscle to inhibit emptying while simultaneously stimulating epithelial secretion to increase filling, leading to significant gallbladder enlargement and cholestasis (Li et al., 2011). This mechanism provides critical evidence for the potential risks of bile-based TCMs. Since these TCMs contain natural TGR5 ligands, their improper use could theoretically result in gallbladder stasis and enlargement, like the adverse effects seen in TGR5-targeted clinical trials. Therefore, to overcome the above-mentioned side effects, it is crucial to develop tissue-specific TGR agonists.

**FIGURE 6:**
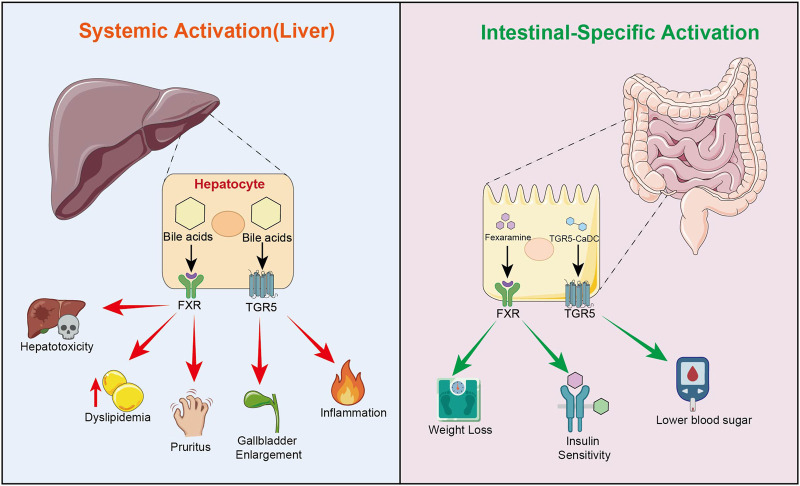
Strategies for tissue-specific receptor activation to minimize systemic toxicity. This schematic contrasts the clinical outcomes of systemic versus localized receptor modulation. Systemic Activation (Liver): Activation of hepatic FXR and TGR5 by bile acids often triggers dose-dependent adverse effects, including hepatotoxicity, dyslipidemia, pruritus, and gallbladder enlargement. Intestinal-Specific Activation: Utilizing intestine-restricted agonists, such as Fexaramine (FXR ligand) and TGR5-CaDC (TGR5 ligand), selectively targets the gut-liver axis. This approach retains significant therapeutic benefits, including weight loss, improved insulin sensitivity, and lower blood sugar, while circumventing the risks associated with direct hepatic activation.

In the latest study, Zhang et al. constructed an intestine-restricted TGR5 agonist-TGR5-CaDC. By covalently linking DCA to a non-absorbable silicon-based carrier (R500), they achieved intestine-targeted delivery of DCA and hepatic “off-target” avoidance. This strategy utilizes the efficient activation of TGR5 on intestinal L cell surfaces by DCA, significantly promoting the secretion of GLP-1. It achieves superior glucose-lowering effects compared to semaglutide in mouse and Bama pig models, while completely avoiding toxic manifestations such as gallbladder dilation, elevated liver enzymes, and systemic inflammatory responses (Zhang et al., 2025). This study not only first confirmed the feasibility and superiority of the “intestine-restricted bile acid activation” strategy, but also provided important inspiration for the modernization of bile-based TCMs: by blocking the systemic absorption pathway of bile acids through structural modification or carrier design while preserving their therapeutic effects via intestinal receptor activation, it may fundamentally solve the “therapeutic-toxicity” double-edged sword problem of bile acids. In the future, precise application of bile-based TCMs may adopt this strategy to construct novel “intestine-targeted, liver-off-target” bile acid delivery systems, thereby maximizing therapeutic efficacy while minimizing tissue-specific toxicity risks (Figure 6).

### 7.1 Mechanistic crosstalk between FXR and TGR5: The antiviral Yin-Yang balance

The therapeutic value of bile-based TCMs lies in the delicate balance between FXR and TGR5 signaling. In viral hepatitis, these two receptors exert opposing effects on the IFN response. Activation of TGR5 via the TGR5-β-arrestin-SRC axis promotes the phosphorylation of signaling proteins that enhance IFN production, thereby initiating innate antiviral immunity (Hu et al., 2019). Conversely, FXR activation can impair antiviral defenses. The FXR-IRF3 interaction suppresses the transcriptional activity of IRF3, leading to reduced IFN expression and potentially facilitating viral persistence (Liang et al., 2024).

**FIGURE 7:**
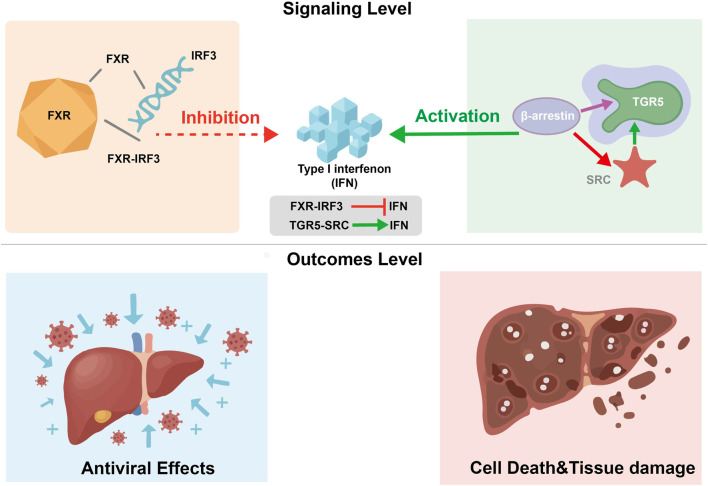
The “Yin-Yang” balance of FXR and TGR5 crosstalk in modulating IFN responses and hepatocyte homeostasis. Figure Legend: This schematic illustrates the molecular competition within the “Yin-Yang” framework of bile acid receptors. Signaling Level: TGR5 activation via the β-arrestin-SRC axis promotes IFN expression, serving as a primary driver of innate immunity. Conversely, the FXR-IRF3 interaction acts as a molecular counterbalance that inhibits IFN transcription to prevent excessive inflammatory responses. Outcomes Level: The biological output of this crosstalk determines the transition between protection and pathology. While balanced signaling facilitates effective Antiviral Effects (left), a disruption of this equilibrium leading to excessive IFN signaling can trigger programmed Cell Death and Tissue Damage (right).

The activation of these receptors is beneficial during the acute phase of metabolic disorders or early viral entry to restore homeostasis and trigger host defense. However, it may become detrimental if the signaling balance is lost. For example, while TGR5-mediated IFN production is necessary for viral clearance, excessive or prolonged IFN signaling can trigger “interferon-mediated cell death” (such as apoptosis or pyroptosis), leading to severe hepatocyte loss and tissue injury (Liang et al., 2024; Hu et al., 2019). Similarly, chronic FXR over-activation by synthetic agonists often results in systemic side effects like pruritus and dyslipidemia, which are not typically observed with the multi-component, milder action of bile-based TCMs (Nevens et al., 2016). This “Yin-Yang” crosstalk ensures that the liver responds flexibly to metabolic and inflammatory stresses, but its disruption can shift the outcome from protection to pathology. The integrated mechanistic landscape of these receptors is summarized in Table 6 and Figure 7.

**TABLE 6:** Risk-benefit analysis of FXR and TGR5 activation in liver diseases.

Feature	Therapeutic advantages	Specific risks and mechanisms	Clinical implications for safety
FXR activation (systemic)	Anti-inflammation, anti-fibrosis, improved lipid/glucose metabolism (Liang et al., 2024; Jiang et al., 2023; Xue et al., 2025). Suppress hepatocarcinogenesis by promoting DNA repair (Huang et al., 2015; Huang et al., 2022).	Dose-dependent hepatotoxicity (Nevens et al., 2016). Tumorigenic controversy: May stimulate cell proliferation in specific human HCC cell lines (Han et al., 2014; Wang et al., 2013) FXR-mediated FGF19 induction has pro-tumorigenic potential (Piglionica et al., 2018).	Benefits require careful dosing. Clinicians should monitor patients for off-target proliferation, especially given the species differences and cell-line specific responses.
TGR5 activation (systemic)	GLP-1 secretion, innate antiviral immunity, anti-inflammation (Hu et al., 2019; Zhang et al., 2025; Zheng et al., 2015). Inhibits HCC cell proliferation in early-stage carcinogenesis (Han et al., 2014; Wang et al., 2013).	Gallbladder enlargement/stasis (Li et al., 2011; Briere et al., 2015). Biliary cancer risk: Overexpression in cholangiocarcinoma and cystic cells may promote tumor cell survival and cyst growth (Ma et al., 2021; Keitel and Haussinger, 2018).	Good for acute viral defense; however, chronic activation needs extreme caution in patients with biliary cancers or polycystic liver disease.
Bile acid accumulation	Natural regulation of bile acid pool, FXR/TGR5 activation (when balanced) (Kim and Fang, 2018)	Direct Hepatocyte Damage: Excessive accumulation of specific bile acids (e.g., high TCA, GCA) causes oxidative stress, ER stress, and triggers apoptosis/necrosis in liver cells (Tian et al., 2021; Yuan et al., 2023).	Bile-based TCMs need balanced components; continuous monitoring of serum bile acid levels is important during treatment to prevent accumulation-induced injury.

## 8 Quality and limitations of clinical evidence

The clinical translation of bile-based TCMs and their components faces several challenges regarding the quality of existing evidence. Most clinical studies on BBP and TUDCA are characterized by small sample sizes and short follow-up durations. For instance, a randomized, double-blind trial of TUDCA in liver cirrhosis included only 60 patients and lasted 6 months (Pan et al., 2013). While such studies demonstrate improvements in surrogate markers, such as ALT, AST, and albumin, they are often underpowered to assess “hard” clinical endpoints like survival rates, incidence of HCC, or liver decompensation (Xiang et al., 2013; Pan et al., 2013).

Furthermore, the comparability of endpoints across different studies remains low. For UDCA, although large cohorts in PBC have been analyzed, systematic reviews indicate that biochemical improvement does not always correlate with a reduction in liver transplant rates or all-cause mortality (Ishibashi et al., 2011; Lammers et al., 2014). Confounding factors, including patient genetic backgrounds and concomitant drug use, often obscure the direct therapeutic benefits (Ishibashi et al., 2011).

The evidence for HDCA is currently even more limited, as its efficacy has primarily been demonstrated in rodent models (Kuang et al., 2023; Xue et al., 2025). While HDCA shows potential in regulating the gut-liver axis and reducing fibrosis in mice, the dose-exposure-response curve and long-term safety in humans remain unknown. Therefore, these animal findings should be framed as exploratory rather than confirmatory (Kuang et al., 2023; Zhong et al., 2023). Future research must prioritize large-scale, multicenter trials using validated surrogate markers (e.g., FibroScan, HVPG) followed by long-term observation of hard clinical outcomes to provide high-confidence evidence for clinical practice (Chunmei et al., 2006; Zhang et al., 2013).

## 9 Regulatory, ethical, and standardization challenges

The use and study of bile-based TCMs face several important issues. These include concerns about animal welfare, how they are regulated, and ensuring consistent quality.

### 9.1 Ethical and conservation concerns

Bear bile, especially from wild or farmed bears, is a sensitive topic. Many international rules, like the Convention on International Trade in Endangered Species of Wild Fauna and Flora (CITES), control or stop its trade (Li et al., 2016; Dutton et al., 2011). Bear farming, where bile is collected from live bears, is seen as cruel by animal welfare groups. This practice causes much suffering to the animals (Dutton et al., 2011). Because of these strong ethical concerns, scientists are looking for other ways to get similar medicines. These include making artificial bile in labs, using bile from other animals like pigs and cows, or finding helpful plant-based ingredients (Li et al., 2016; Feng et al., 2009). These efforts aim to provide effective treatments without harming endangered species or causing animal cruelty.

### 9.2 Quality control and regulatory oversight

Different countries have different rules for bile-based TCMs. For example, in China, the Chinese Pharmacopoeia sets strict rules for animal-derived bile powders (Chinese Pharmacopoeia Commission, 2010). These rules cover where the bile comes from, how it is made, how to identify it, and how much active ingredient it should have. They also set limits for harmful substances. Following Good Manufacturing Practice (GMP) is very important. GMP ensures that these medicines are made in a consistent way, are safe, and are of good quality. However, making sure all producers meet these GMP standards can be difficult, especially for traditional products (Li et al., 2016).

### 9.3 Managing compositional variability

The makeup of bile acids can differ a lot. This depends on the animal, where it lives, what it eats, and how the bile is processed (Li et al., 2016; Yan et al., 2022; Qiao et al., 2011). For example, bear bile has more TUDCA and TCDCA, while pig bile has more HDCA (Qiao et al., 2014; Yan et al., 2022; Yan et al., 2017). Cow bile contains more GCA and TCA (Yan et al., 2022; Yan et al., 2016). These differences make it hard to set standard doses and predict how well the medicine will work. To solve this, researchers use advanced tests, such as HPLC-MS and NMR, to carefully check the bile acid content (Yan et al., 2022; Changsheng et al., 2012). The goal is to create clear standards for quality. This will help doctors use these medicines more precisely and compare results better (Li et al., 2016; Qiao et al., 2014; Yan et al., 2022). Making artificial bile with a fixed composition is a good way to reduce this natural variability and ensure consistent quality (Li et al., 2016).

## 10 Future perspectives and clinical strategies

Future trials of bile-based TCMs must adopt large, multicenter, long-term designs, ideally adaptive or platform protocols, validating surrogate markers (FibroScan, HVPG, ELF) before proceeding to hard endpoints (decompensation, transplant, death). A paramount priority for future clinical design is the distinction between intestinal and hepatic receptor activation. Future research should emphasize the development of intestine-restricted agonists that utilize the gut-liver axis, specifically triggering GLP-1 secretion from intestinal L-cells, while explicitly avoiding systemic absorption. A successful example of this strategy is TGR5-CaDC, an intestine-restricted TGR5 agonist that effectively improves glycemic control in animal models while completely avoiding gallbladder dilation and systemic inflammatory responses. This approach is essential because, while bile acid analogs are therapeutically potent, their direct activation of intrahepatic receptors frequently triggers dose-dependent hepatotoxicity, cholestasis, and pruritus.

Furthermore, clinical trials must investigate the molecular mechanisms of DILI related to bile acid nutritional homeostasis. Many drug candidates fail due to idiopathic hepatotoxicity, which likely stems from their inadvertent interference with the hepatic bile acid pool or the aberrant activation of intrahepatic receptors. Therefore, monitoring dynamic bile acid content and receptor activation profiles during early clinical phases is a highly actionable measure to detect early homeostatic imbalances. For instance, while OCA is effective in improving biochemical markers in PBC patients, its clinical use is often limited by side effects such as severe pruritus and dyslipidemia, which are linked to its systemic activation of FXR. Stratified randomization by etiology, fibrosis stage and metabolic comorbidities, together with pre-specified subgroup analyses for T2DM and chronic kidney disease (CKD), is essential to evaluate how different populations respond to these site-specific therapies. Finally, the integration of exposure-response modelling with FXR/TGR5 pathway activation and bile-acid metabolomics will furnish the robust evidence required for high-confidence, clinically applicable conclusions.

## 11 Conclusion

Bile-based TCMs represent a sophisticated interface between millennia of clinical experience and modern molecular pharmacology. Our review establishes that the traditional “Yin-Yang” framework is rooted in quantifiable biological parameters, specifically the hydrophilic-hydrophobic balance and receptor activation thresholds. The therapeutic “Yang” effects, driven by components like TUDCA and UDCA, are characterized by their low hydrophobicity and ability to maintain homeostatic signaling via FXR and TGR5 without triggering intrahepatic toxicity. Conversely, the “Yin” potential of these medicines is defined by hydrophobic conjugates like TCA, GCA, and GDCA. When these components exceed critical concentration thresholds (50–100 μM), they shift from signaling molecules to mediators of apoptosis, fibrosis, and immune suppression. This dual nature provides a rigorous scientific explanation for why these substances can be both highly effective and potentially toxic, depending on their precise chemical composition and the pathological context of the liver.

One of the primary advantages of bile-based TCMs over modern single-molecule drugs, such as OCA, is their multi-target capability. While single-target synthetic agonists often trigger dose-dependent side effects like severe pruritus and dyslipidemia through systemic receptor over-activation, the natural mixture of bile acids in TCMs regulates the gut-liver axis more comprehensively. This holistic regulation facilitates the simultaneous correction of metabolic disorders, inflammation, and immune imbalance. However, this complexity also demands a more rigorous approach to standardization. The inherent variability in bile acid profiles across different animal sources remains a significant challenge, necessitating the use of advanced analytical techniques like HPLC-MS and NMR to ensure batch-to-batch consistency and clinical safety.

The successful translation of these traditional therapies into modern medicine also depends on resolving the ethical and regulatory dilemmas associated with animal-derived products. The shift toward high-quality artificial bear bile and the identification of plant-based alternatives represent a necessary evolution in the field. These substitutes must be validated not only for their chemical similarity but also for their functional equivalence in replicating the synergistic therapeutic effects of the original formulations. Adopting strict GMP standards and international conservation policies is essential to ensure that bile-based therapies are both ethically acceptable and scientifically standardized for global use.

Ultimately, the evolution of bile-based therapies signifies a transition toward “Precision TCM.” This paradigm shift requires moving beyond empirical traditional use and toward a strategy defined by tissue-specific signaling and molecular monitoring. By focusing on intestine-restricted receptor activation and integrating exposure-response modeling with bile-acid metabolomics, we can maximize the therapeutic window of these ancient medicines. In summary, through the integration of rigorous clinical trial design and deep mechanistic understanding, bile-based TCMs can be transformed into safe, evidence-based, and highly effective treatments that address the growing global burden of chronic liver diseases.

## Glossary

**ALT**: alanine aminotransferase
**AST**: aspartate aminotransferase
**BBBP**: biotransformed bear-bile powder
**BBP**: bear-bile powder
**BSEP**: bile-salt export pump
**CBP**: cow-bile powder
**CDCA**: chenodeoxycholic acid
**CFLD**: cystic fibrosis-related liver disease
**CHB**: chronic hepatitis B
**CHF**: cholestatic hepatic fibrosis
**CKD**: chronic kidney disease
**COX-2**: cyclo-oxygenase-2
DCA: deoxycholic acid
**DILI**: drug-induced liver injury
**ETV**: entecavir
**FXR**: Farnesoid X receptor
**GCA**: glycocholic acid
**GCDCA**: glycochenodeoxycholic acid
**GDCA**: glycodeoxycholic acid
**GHDCA**: glycohyodeoxycholic acid
**HCA**: hyocholic acid
**HDCA**: hyodeoxycholic acid
**HCC**: hepatocellular carcinoma
**HPLC-ELSD**: high-performance liquid chromatography with evaporative light-scattering detection
**IRI**: ischemia-reperfusion injury
**LDL-C**: low-density lipoprotein cholesterol
**LPS**: lipopolysaccharide
**MASH**: metabolic-dysfunction-associated steatohepatitis
**MASLD**: metabolic-dysfunction-associated steatotic liver disease
**NASH**: non-alcoholic steatohepatitis
**NK**: natural killer (cell)
**NO**: nitric oxide
**OCA**: obeticholic acid
**PBP**: pig bile powder
**PBC**: primary biliary cirrhosis
**PSC**: primary sclerosing cholangitis
**SCFA**: short-chain fatty acid
**STAT3**: signal transducer and activator of transcription 3
**TAF**: tenofovir alafenamide fumarate
**TCA**: taurocholic acid
**TCDCA**: taurochenodeoxycholic acid
**TDCA**: taurodeoxycholic acid
**TGR5**: Takeda G protein-coupled receptor 5
**THDCA**: taurohyodeoxycholic acid
**TUDCA**: tauroursodeoxycholic acid
**UDCA**: ursodeoxycholic acid
